# Application of Electrical Capacitance Method for Prediction of Plant Root Mass and Activity in Field-Grown Crops

**DOI:** 10.3389/fpls.2018.00093

**Published:** 2018-02-01

**Authors:** Imre Cseresnyés, Katalin Szitár, Kálmán Rajkai, Anna Füzy, Péter Mikó, Ramóna Kovács, Tünde Takács

**Affiliations:** ^1^Institute for Soil Sciences and Agricultural Chemistry, Centre for Agricultural Research, Hungarian Academy of Sciences, Budapest, Hungary; ^2^Institute of Ecology and Botany, Centre for Ecological Research, Hungarian Academy of Sciences, Vácrátót, Hungary; ^3^Agricultural Institute, Centre for Agricultural Research, Hungarian Academy of Sciences, Martonvásár, Hungary

**Keywords:** arbuscular mycorrhizal fungi, field monitoring, maize, phenology, root activity, root capacitance, soybean

## Abstract

The root electrical capacitance (C_*R*_) method is suitable for assessing root growth and activity, but soil water content (SWC) strongly influences the measurement results. This study aimed to adapt the method for field monitoring by evaluating the effect of SWC on root capacitance to ensure the comparability of C_*R*_ detected at different SWC. First a pot experiment was conducted with maize and soybean to establish C_*R*_–SWC functions for the field soil. Ontogenetic changes in root activity were monitored under field conditions by simultaneously measuring C_*R*_ and SWC around the roots. The C_*R*_ values were normalized using SWC data and experimental C_*R*_–SWC functions to obtain C_R_^*^, the comparable indicator of root activity. The effect of arbuscular mycorrhizal fungi (AMF) inoculation on the C_R_^*^ and biomass of field-grown soybean was investigated. The pot trial showed an exponential increase in C_*R*_ with SWC. C_*R*_–SWC functions proved to be species-specific. C_*R*_ showed strong correlation with root dry mass (*R*^2^ = 0.83–0.87). The root activity (C_R_^*^) of field-grown crops increased until flowering, then decreased during maturity. This was consistent with data obtained with other methods. AMF inoculation of soybean resulted in significantly higher C_R_^*^ during the late vegetative and early flowering stages, when destructive sampling concurrently showed higher shoot biomass. The results demonstrated that the root capacitance method could be useful for time course studies on root activity under field conditions, and for comparing single-time capacitance data collected in areas with heterogeneous soil water status.

## Introduction

Due to the inherent methodological difficulties associated with monitoring root growth and activity in the soil, there has been continuous interest in the use and development of simple, rapid *in situ* methods providing information on the root status without damaging the plant (Milchunas, 2012). One such non-intrusive technique is based on the electrical properties of the root system. The root electrical capacitance (C_*R*_) method was first applied by Chloupek (1972) in various monocot and dicot crop species, using a capacitance meter with low-voltage (1V) alternating current (1,000 Hz AC). Chloupek found, that the C_*R*_ measured between a ground electrode (inserted in the soil) and a plant electrode (attached to the stem) showed positive correlation with root dry mass (RDM), root length (RL) and root surface area (RSA).

C_*R*_ arises due to the active polarization of root membranes by AC, which decreases the magnitude and shifts the phase of the current signal. The first biophysical model, proposed by Dalton (1995), considers the root system as a group of parallel-connected cylindrical condensers, in which the polarized root membranes (dielectric in the capacitor) separate the highly conductive root sap from the highly conductive hydroponic or soil solution. The capacitance detected (C_*R*_) is proportional to the surface area of the charge-storing membranes. Rajkai et al. (2005) and Dietrich et al. (2013) recognized that the rooting substrate also has an electrical capacitance. They formulated a “two-dielectric capacitor model” that consists of series-connected root and a soil dielectric media with different relative permittivity (ε_*r*_). Authors stated that, “provided the capacitance of the root tissue is much smaller than that of the rooting substrate, the capacitance of the plant–substrate system is determined by the root tissue.” In a revised model, Dietrich et al. (2012, 2013) emphasized that “the root tissue is a continuous dielectric, and the capacitances of tissues along an unbranched root are connected in series, and the whole root system in parallel.” Ellis et al. (2013a) verified the role of the tissue density and ε_*r*_ of the root cortex in determination of C_*R*_.

The generalization of the capacitance technique is limited due to the sensitivity of C_*R*_ to external factors, such as soil water content (SWC), texture and ionic composition, and the position of the plant electrode (Dalton, 1995; Ozier-Lafontaine and Bajazet, 2005; Ellis et al., 2013b). Kormanek et al. (2016) confirmed that the surface area and shape of the soil electrode strongly affected the correlation between C_*R*_ and root system parameters. Nevertheless, under standardized soil conditions and with constant height of plant electrode above the substrate surface, the method adequately estimates the root system size (RSS) (Postic and Doussan, 2016). The great advantage of the capacitance method is that, since electric current passes almost exclusively through absorbing root surfaces and not through non-absorbing (suberized) root segments, C_*R*_ has potential for assessing “functional root extent,” discerning root activity from a measure of RSS (Čermák et al., 2006; Cseresnyés et al., 2016a). Unlike other widely used techniques, capacitance method measures root activity including root hairs.

The C_*R*_ method was used to develop specific calibration relationships with reasonable predictive ability to obtain an absolute measurement of root traits for a given plant grown in a given soil (Preston et al., 2004; McBride et al., 2008; Tsukahara et al., 2009; Pitre et al., 2010). The technique was also applied without specific calibration for the comparison of RSS when studying plant responses to environmental stresses (e.g., herbicide treatment, heavy metal contamination, weed competition) and arbuscular mycorrhizal fungi (AMF) colonization, and to monitor cultivar-specific differences in root growth dynamics (Vamerali et al., 2009; Cseresnyés et al., 2012, 2013, 2016a,b). Chloupek et al. (2010) emphasized that “C_*R*_ data are only comparable for plants of the same species, grown in the same substrate at the same moisture level.” The non-intrusive capacitance method is appropriate for repeated evaluation of the same plant population at different ontogenetic phases, provided that SWC in the pots is identical at each measurement time. Under field conditions, the temporal changes in SWC preclude continuous root monitoring in most cases, so field-grown plants can only be compared based on C_*R*_ at a single date with identical SWC. In this manner, the C_*R*_ method was successfully applied in the field to investigate root traits of barley and wheat genotypes (Nakhforoosh et al., 2014; Svačina et al., 2014; Heřmanská et al., 2015) and to characterize varietal differences in their drought tolerance and water use efficiency (Chloupek et al., 2010; Středa et al., 2012).

Taking changes in SWC into consideration in C_*R*_ measurement and data evaluation may provide an opportunity for the continuous monitoring of root activity under field conditions, irrespective of soil water conditions. Soils provide a large electrical capacitance (C_*S*_), the magnitude of which is determined not only by physical and chemical soil properties but also by SWC (Hilhorst, 1998). Though C_*S*_ is easy to detect at any SWC using two ground electrodes identical to those used for C_*R*_ measurement, knowledge of C_*S*_ cannot provide information on the capacitance exhibited by the root system (see the aforementioned two-dielectric capacitor model). The root–soil–electrode network consists of resistance and capacitance elements variously associated and interfering, leading to very complicated electrical behavior (Ozier-Lafontaine and Bajazet, 2005). Therefore, to the best of our knowledge, there is as yet no mathematical model that considers changes in SWC when evaluating C_*R*_.

For this reason, it was aimed to investigate the relationship between the detectable C_*R*_ and SWC in pots, followed by a time-course study to test the applicability of the results under field conditions. (i) A pot trial was designed with maize and soybean plants to develop regression models to characterize the influence of SWC on C_*R*_, and to evaluate the effect of plant species and RSS (plant age) on the regression parameters. (ii) It was planned to carry out repeated measurements on C_*R*_ and SWC around the roots of field-grown maize and soybean throughout the vegetation season. It was hypothesized that, using the empirical models obtained from the pot experiment, the measured C_*R*_ values could be transformed (on the basis of SWC) into an “apparent” root electrical capacitance, C_R_^*^, which would be detectable in fully water-saturated soil. In this manner, the effect of SWC on capacitance measurement could be eliminated, ensuring the comparability of field data collected at different times (with different SWC) during the growing season. (iii) Seasonal changes in C_R_^*^ (as an indicator of root activity) were investigated in field-grown crops in relation to plant ontogeny in order to check the usability of our approach for field monitoring studies. (iv) Root colonization of AM fungi is known to increase the water and nutrient uptake capacity and biomass production of the host plant; the hyphal contribution to absorptive RSA proved to be detectable by monitoring C_*R*_ in pot experiments (Cseresnyés et al., 2013). Leguminous soybean is a typical host for AMF symbionts, so two treatments (control and inoculated) were designed to monitor the effect of AMF colonization on plant root activity (C_R_^*^) under field conditions.

The general purpose of the research was to adapt the C_*R*_ method for use in the case of varying SWC, thus affording the possibility to monitor root activity and to detect treatment differences *in situ* in the field.

## Materials and methods

### Pot experiment

#### Plant cultivation

Seeds of maize (*Zea mays* L., cv. Mv343) and soybean (*Glycine max* L. Merr., cv. Aliz; maturity group 0; indeterminate growth habit) were germinated on wet paper towels in Petri dishes in darkness at 24°C for 2 days. The seedlings were planted into 2.6 dm^3^ cylindrical plastic pots containing 3.5 kg of air-dried, coarsely sieved chernozem soil collected from the Martonvásár nursery (Table 1). Last year's dead roots and other plant materials were also removed by sieving. Before planting, the soil was irrigated with tap water to field capacity by placing the pots on a balance (±1 g). Seeds were planted twice a week, with a single maize or soybean seed in each pot, until there were 15 replicates of each species (49-day planting period) to obtain a wide range of RSS with one harvest at the end of the experiment. The plants were cultivated in a growth chamber at 28/18°C day/night temperature, 16/8 h photoperiod, 600 μmol m^−2^ s^−1^ PAR and 50–70% relative humidity, watering the soil to field capacity every other day.

**Table 1:** Physicochemical properties of soil used for pot experiment and field trial.

**Soil property**	**Value**
Sand/silt/clay content [%]	33.8/42.3/23.9
pH_H2O_/pH_KCl_	7.69/7.08
Cation exchange capacity (CEC) [mmol 100 g^−1^]	17.34
CaCO_3_ content [%]	1.58
Humus content [%]	3.17
N/P/K content [mg kg^−1^]^(a)^	1933/371/402
Bulk density [g cm^−3^]	1.39
Saturation water content [cm^3^ cm^−3^]	0.476
Field capacity [cm^3^ cm^−3^]	0.305
Permanent wilting point [cm^3^ cm^−3^]	0.097

(a) *Total organic and mineral N content; ammonium lactate acetate-extractable P and K*.

Three days after the last planting, when the youngest plants emerged, water was withheld until SWC decreased to near wilting point (~10 w/w%). Volumetric SWC was measured daily with a TDR instrument (Trase1; Soilmoisture Equip. Co., Santa Barbara, CA, USA) calibrated to this soil using a 15-cm-long waveguide set. A little water was added to the pots if necessary to maintain the required SWC. Thirteen days after the last planting, SWC was adjusted to slightly above the wilting point in all 30 pots (plant age: 13–62 days).

#### Root electrical capacitance (C_*R*_) measurement and plant harvest

The SWC around the roots was detected by TDR in each pot, after which all the plants were immediately subjected to C_*R*_ measurement (first SWC–C_*R*_ data pair). Parallel capacitance (C_*R*_) was detected with an Agilent U1733C handheld LCR meter (Agilent Techn. Co. Ltd., Penang, Malaysia) at 1 kHz and 1 V AC. The ground electrode was an 18-cm-long stainless steel rod (6.3 mm i. d.) inserted vertically to 15 cm depth in the soil, 6 cm away from the stem. The plant electrode was a clamp fixed 15 mm above the soil surface through a 5-mm-wide aluminum strip that bent the stem (Cseresnyés et al., 2016a). Electrocardiograph paste was used under the strip to ensure good electric connection (Rajkai et al., 2005). The electrodes were left in place throughout the experiment.

Soon after the first detection of SWC and C_*R*_, 100 mL of tap water was poured over the soil in each pot, and a second measurement was made an hour later (increase in soil ion content by tap water irrigation had a negligible effect on C_*R*_ measurement due to the much higher electrical capacitance and conductance of chernozem soil compared to those of roots). The irrigation and measurement steps were repeated several times until SWC approached field capacity, when the pot drain holes were closed to prevent water leakage and the procedure was continued until the soil became nearly water-saturated (nine SWC–C_*R*_ data pairs). Right after the drain holes were reopened, the pots were placed in water-filled containers (water level at soil surface level) overnight in order to saturate the soil with water, after which the last measurements were performed (tenth SWC–C_*R*_ data pair). The parallel capacitance of the soil (C_*S*_) in the pots was also detected between two identical ground electrodes inserted in the substrate at a distance of 6 cm after each C_*R*_ reading.

All the plants were harvested at the end of the experiment. After cutting off the shoots at the soil surface, the soil was thoroughly washed off the root systems with running water over a 0.2-mm mesh sieve followed by the root flotation. The roots were placed between paper towels to remove the excess of water from root surfaces, then were instantly put in a pre-heated (70°C) oven to dry to constant weight and determine RDM (±0.001 g).

#### Data analysis

The relationship between RDM and C_*R*_ measured at wilting point and field capacity was evaluated for both species by simple regression analysis in order to verify the validity of the linear relationship at highly different SWC. All measured SWC values were converted to relative water saturation (θ_rel_) based on the saturation water content (0.476 cm^3^ cm^−3^). A C_*R*_-θ_rel_ function was calculated for each of the 15 maize and 15 soybean specimens; R^2^ was determined using the ln-transformed C_*R*_ variables. For a given specimen, the C_*R*_ measured in water-saturated soil (θ_rel_ = 1) was considered as the apparent (saturation) capacitance, C_R_^*^; then all measured C_*R*_ values were divided by C_R_^*^ to obtain the relative capacitance, C_rel_ for each θ_rel_ value. Linear regressions were used to examine the relationship between ln-transformed C_rel_ and θ_rel_ for each specimen.

Next, the effect of plant age and species identity on the C_rel_-θ_rel_ functions was investigated for the two species, using linear mixed effect models (LME) where C_rel_ was a ln-transformed response variable, θ_rel_, species and plant age (days) were explanatory variables, and individuals as a categorical random effect. As the effect of species was significant, separate LME analysis was conducted for each species to obtain species-specific C_rel_-θ_rel_ functions that could be used to calculate C_R_^*^. In these analyses, θ_rel_ was considered as the only explanatory variable (as plant age had no significant effect on C_rel_ in the previous analysis) and individuals as a categorical random effect. Statistical analyses were performed using the nlme (Pinheiro et al., 2012) and MuMIn packages (Bartoń, 2015) in R ver. 2.15.2 environment (R Core Team, 2012).

### Field trials

#### Site description

Field studies were conducted in the Martonvásár nursery (N47°18′41″, E18°46′48″, 109 m asl.) in 2015. The site is characterized by haplic chernozem soil (Table 1) and a continental climate with mean (1985–2014) annual temperature of 10.9°C and annual precipitation of 554 mm, 354 mm of which falls during the growing season (April–October). In 2015 the annual rainfall was 18% lower (457 mm) than the long-term average, with normal temperature conditions. Though the total amount of rain during the growing season was completely normal (354 mm), it showed monthly anomalies with a dry April (18% of the mean amount) and July (39%), and a rainy September (180%) and October (301%).

#### Field trial A–maize

The field experiment was designed to examine the performance of the Hungarian maize hybrid, Mv343 (maturity FAO 360) under organic growing conditions on a field area of 0.2 ha (density: 70,000 plants/ha; row distance: 0.76 m; plant distance: 0.19 m). The seeds were sown on 23 April 2015 and harvested on 5 October (growing period: 165 days after sowing, DAS). Weeding was done mechanically (DAS 24 and 38) and no chemicals were used.

Three replicates of 5 m long row-segments were selected randomly for field measurements. SWC and C_*R*_ were recorded (with the same instruments as used in the pot experiments) on 10 occasions during the growing season, from 29 May (DAS 36) to 1 October (DAS 161). No earlier measurement was made because of late seedling emergence on DAS 20–23 due to an extended dry period, when only 39 mm rainfall (27% of the long-term mean) was recorded between 1 February and 19 May. For each measurement, 16 plants were randomly selected from each row-segment. First SWC around the roots was detected by inserting the 15-cm-long TDR waveguide 6 cm from the plant stem, followed by measuring C_*R*_ in the same manner as described in the pot experiment. The plant and ground electrodes were removed after the C_*R*_ reading. On each day of measurement, the phenological stages of the plants were determined according to the BBCH scale Meier, 2001). No destructive plant sampling was conducted either during or at the end of the field trial.

#### Field trial B–soybean

Six 4 × 5 m plots were established after soil tillage, separated by 1-m-wide aisles. Three plots were designated as controls (*CON*), while the others (*INO*) were inoculated with a commercial AMF inoculum “Symbivit” (Symbiom Ltd., Lanskroun, Czech Republic) containing the propagules of six AMF strains. Three kilogram of inoculum per plot was rotated into the upper 25 cm soil layer. The seeds of soybean cultivar Aliz were hand-sown (density: 200,000 plants/ha; row distance: 0.5 m; plant distance: 0.1 m) on 5 May 2015 and harvested on 16 October (DAS 164). Weeds were controlled by hand-hoeing as often as was necessary and no chemicals were used in compliance with organic farming.

SWC and C_*R*_ were measured on ten occasions during the vegetation season, between 13 June (DAS 39) and 14 October (DAS 162). No earlier measurement was made due to late seedling emergence (DAS 26–28) caused by drought (see above). On each measurement day, 16 plants were randomly selected from each *CON* and *INO* plot (not from the edges), after which SWC and C_*R*_ were detected in the same manner as described for the maize crop. The SWC measurements only covered the 0–15 cm soil layer penetrated by the ground electrode attached to the LCR meter, although part of the soybean root system is able to reach much deeper soil depths. However, variations in SWC have been shown to influence the C_*R*_ value detected via the alteration of contact resistance between the ground electrode and the soil but not through the altered root–soil contact (Ellis et al., 2013b). Dietrich et al. (2013) also proved, that the water status of the topsoil is the major constituent in the reliability of C_*R*_ measurements. The plant phenological stages were identified using the BBCH scale (Meier, 2001).

Plants were destructively sampled only four times in the course of the experiment (DAS 48, 71, 99, and 135; 6 plants per plot) and at the final harvest (DAS 164; 10 plants per plot) to reduce soil disturbance. Randomly selected plants were carefully dug out (~25 cm depth) with soil adhering to the roots. The shoots were cut off, then oven-dried at 70°C to constant weight to determine the shoot dry mass (SDM). Grains were also weighed in samples from the last harvest. RDM was not determined, since the isolation of intact root systems (particularly from deeper soil layers) was thought to be impossible in the field. A sample of fine roots was taken to investigate AMF colonization (Cseresnyés et al., 2013). The fine-root samples were washed, cleared and stained with lactic acid–aniline blue (Phillips and Hayman, 1970), after which the intensity (M%) of AMF colonization was investigated microscopically according to the method by Trouvelot et al. (1986).

#### Data analysis

All the C_*R*_ data were transformed into C_R_^*^ using the θ_rel_ values associated with the relevant C_*R*_ readings, according to the empirical formula obtained from the pot experiment. Changes in C_R_^*^ during plant ontogeny were investigated.

Data were analyzed with Statistica software (ver. 12, StatSoft Inc., OK, USA). The unpaired *t*-test or one-way ANOVA with the Tukey–Kramer post-test was used to compare the θ_rel_, C_R_^*^, SDM and AMF colonization (M%) data. In case of significantly different SDs of the compared groups (determined by F-test or Bartlett test), Welch's *t*-test or the Kruskal–Wallis test with Dunn's post-test were applied. Statistical significance was accepted at *p* < 0.05. The relationship between SWC and C_*R*_ was analyzed by the simple regression method (*p* < 0.05).

## Results

### Pot experiment

**Figure 1:**
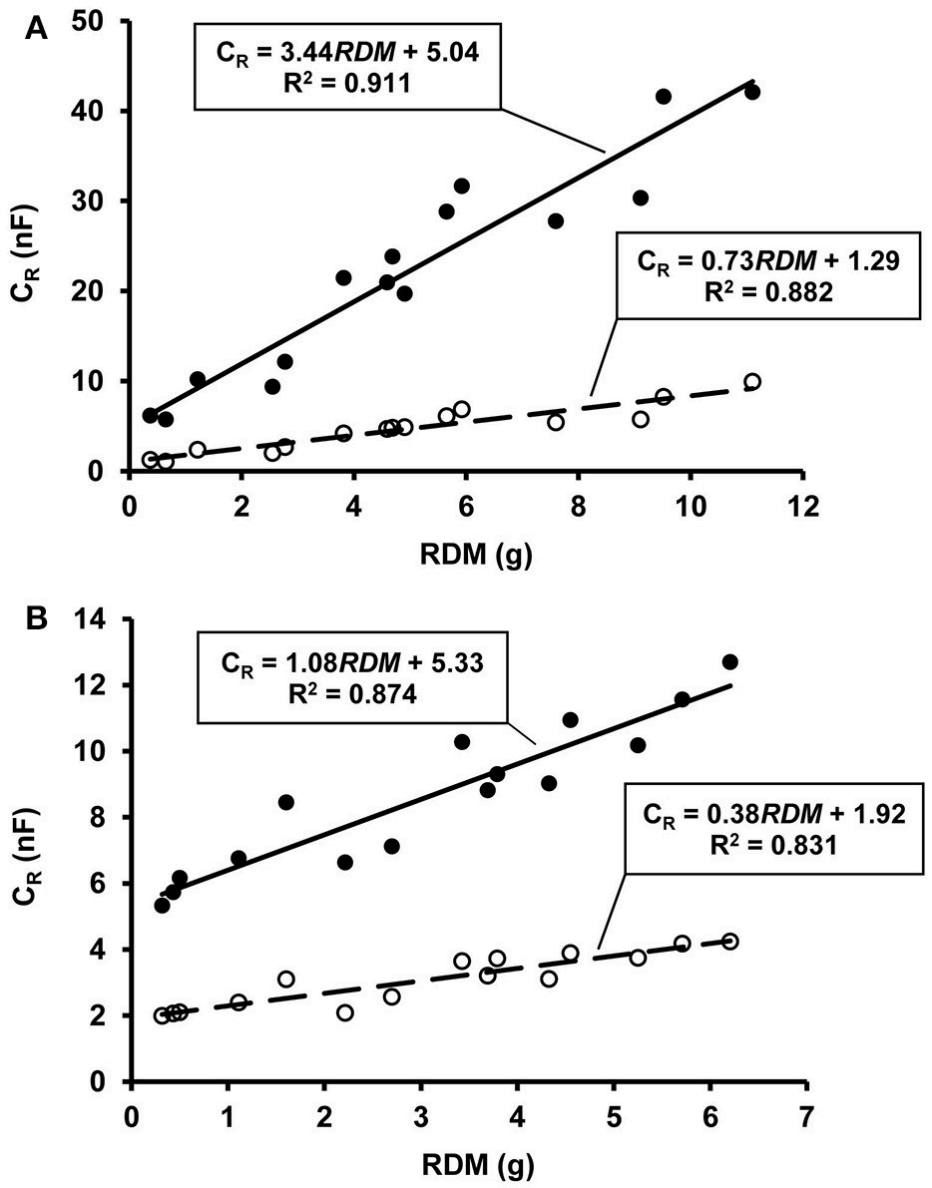
**(A)** Relationship between root electrical capacitance (C_*R*_ in nanofarads, nF) and root dry mass (RDM) in maize plants at permanent wilting point (symbol ◦ with dashed line) and total water saturation of soil (symbol • with solid line), based on pot experiment data. **(B)** The same relationships for soybean plants.

The C_*R*_ value increased at an increasing rate with rising SWC. The oldest maize and soybean specimens reached a maximum C_*R*_ of 42.1 nF and 12.7 nF, respectively, in the fully water-saturated soil. The harvested plants had RDM values ranging from 0.37 to 11.10 g for maize and from 0.32 to 6.21 g for soybean. Regression analysis showed strong linear relationships between RDM and the C_*R*_ (*n* = 15; *R*^2^ = 0.831–0.911; *p* < 0.01) detected either at wilting point or in saturated soil (Figures 1A,B). These results confirmed that C_*R*_ could be used to predict RDM not only at soil moisture levels corresponding to field capacity, at which the C_*R*_ method is conventionally used, but also in the case of extremely low or high SWC. Soil capacitance (C_*S*_) increased with θ_rel_ according to the equation C_*S*_ = 350.3$\cdot \theta_{\text{rel}}^{1.086}$ (*n* = 300; *R*^2^ = 0.934; *p* < 0.01), reaching 346.3 ± 14.2 nF (mean ± SD; *n* = 30) in water-saturated soil. C_*S*_ was at least an order of magnitude higher than the corresponding C_*R*_ at any SWC status.

**Figure 2:**
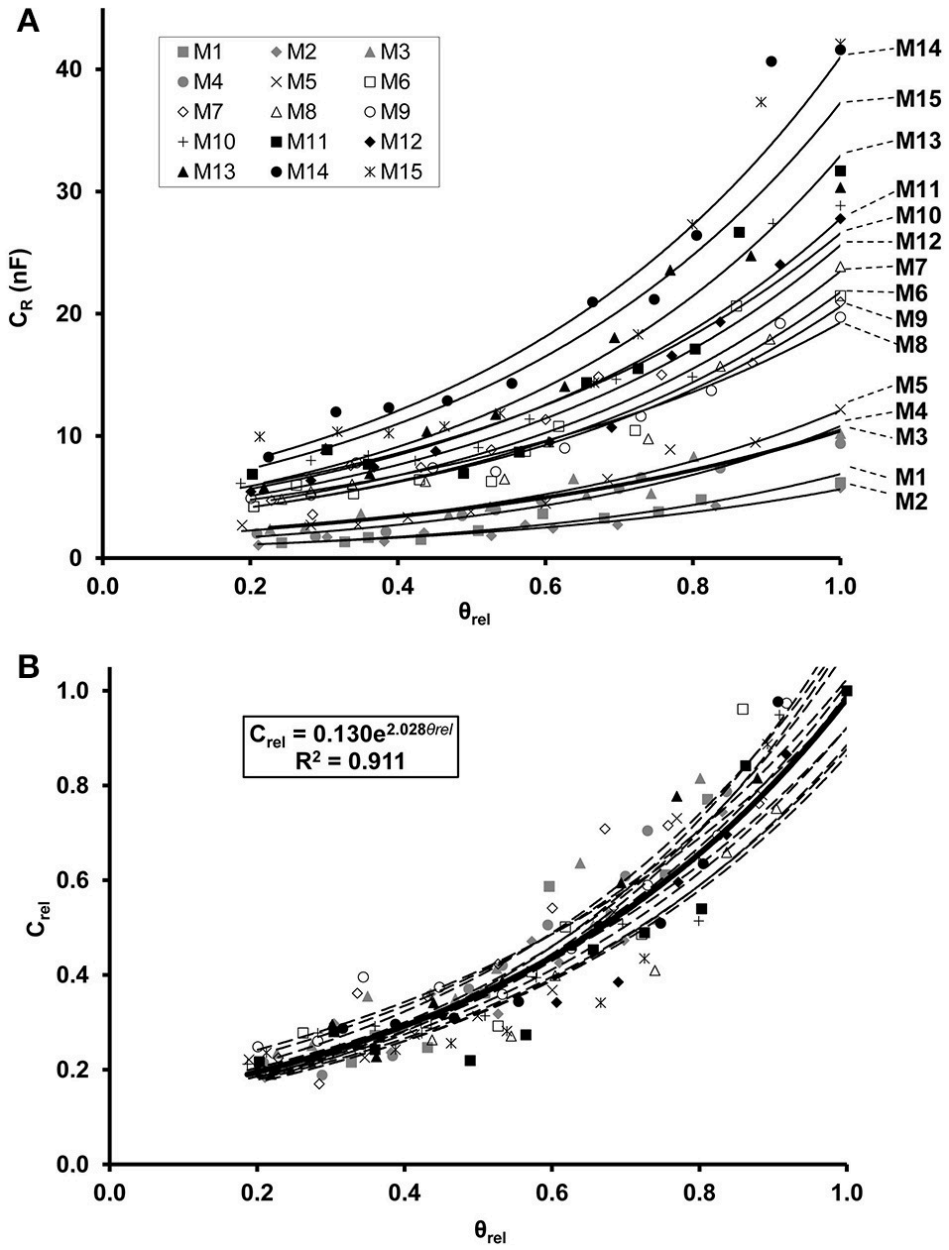
**(A)** Exponential relationship between root electrical capacitance (C_*R*_ in nanofarads, nF) of maize plants and relative water saturation of soil (θ_rel_). Plants M1–M15 in order of increasing root dry mass. **(B)** Exponential relationship between relative root electrical capacitance (C_rel_) and relative water saturation of soil (θ_rel_). C_rel_ is the ratio of C_*R*_ to the root capacitance C_R_^*^ measured in water-saturated soil (θ_rel_ = 1) for the given plant. The regression equation was obtained from the model fitted to the whole data set (*n* = 150; thick solid line).

**Figure 3:**
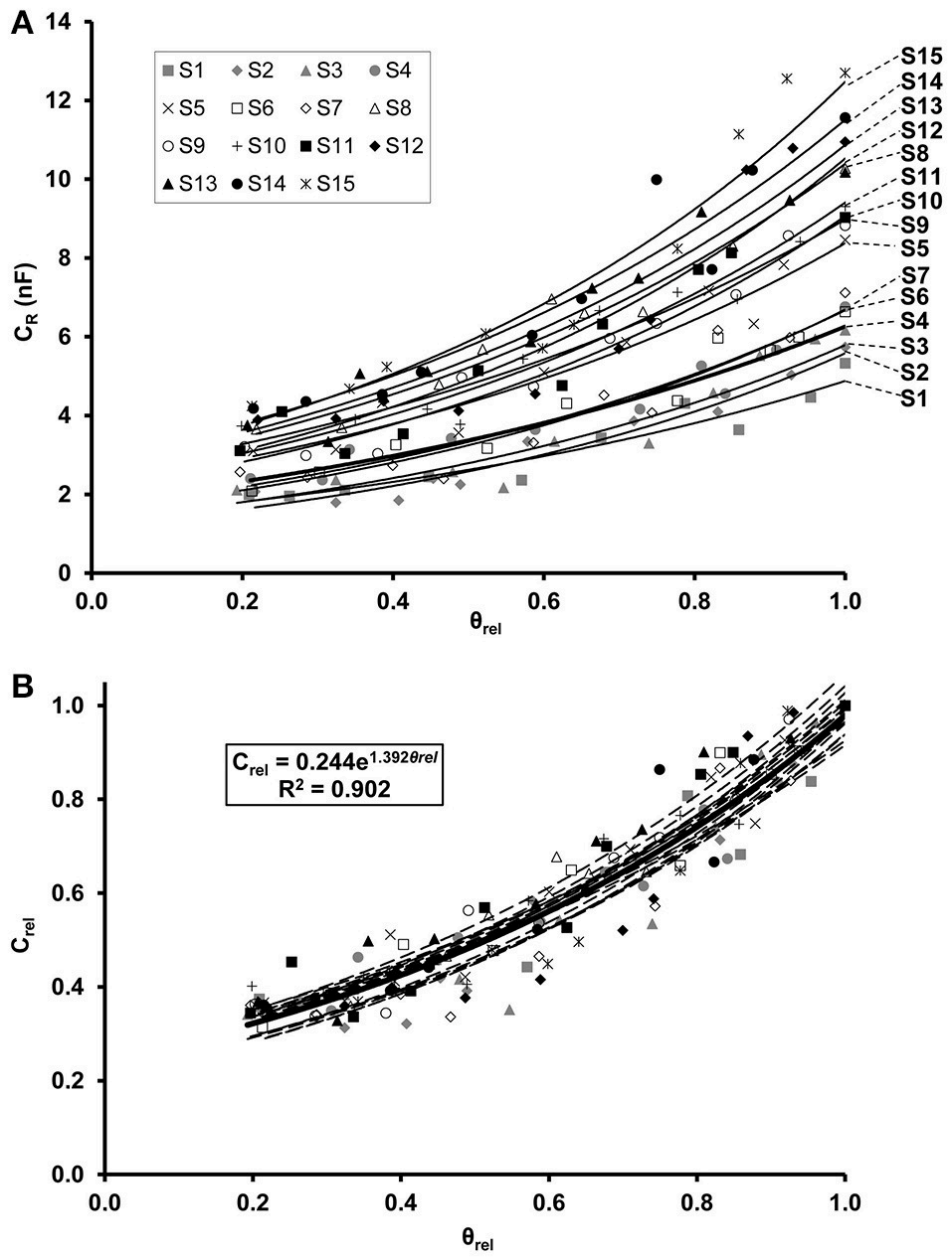
**(A)** Exponential relationship between root electrical capacitance (C_*R*_ in nanofarads, nF) of soybean plants and relative water saturation of soil (θ_rel_). Plants S1–S15 in order of increasing root dry mass. **(B)** Exponential relationship between relative root electrical capacitance (C_rel_) and relative water saturation of soil (θ_rel_). C_rel_ is the ratio of C_*R*_ to the root capacitance C_R_^*^ measured in water-saturated soil (θ_rel_ = 1) for the given plant. The regression equation was obtained from the model fitted to the whole data set (*n* = 150; thick solid line).

Regression analysis demonstrated an exponential increase in C_*R*_ in relation to θ_rel_ for each maize (Figure 2A) and soybean (Figure 3A) plant. Linear relationships (*p* < 0.01) were found between the ln-transformed C_*R*_ data and θ_rel_ with adjusted R^2^ values of 0.854–0.959 and 0.877–0.958 for maize and soybean, respectively. All the C_*R*_ values were divided by the corresponding saturation capacitance (C_R_^*^) to obtain C_rel_ values. Linear regression showed that the relationship between C_rel_ and θ_rel_ could be expressed by the exponential formula C_rel_ = a·e^*b*·θrel^ with computed values of parameters a and b of 0.115–0.170 and 1.75–2.24, respectively, for maize (Figure 2B), and of 0.208–0.273 and 1.24–1.54, respectively, for soybean (Figure 3B).

The LME analysis revealed no significant effect of plant age on C_rel_-θ_rel_ functions, whereas the slope and y-intercept proved to be significantly influenced by the species (Table 2). Separate LME analyses for the two species also demonstrated insignificant effects of plant age on C_rel_-θ_rel_ functions (Table 2), resulting in overall, species-specific relationships of,

$$C_{\text{rel}}=0.130\cdot {\text{e}}^{2.028\cdot \theta \text{rel}}(R^{2}=0.911)$$

for maize, and

$$C_{\text{rel}}=0.244\cdot {\text{e}}^{1.392\cdot \theta \text{rel}}(R^{2}=0.902)$$

for soybean (Figures 2B, 3B). If C_*R*_/C_R_^*^ is substituted for C_rel_, equated with the right-hand side of Equations 1, 2 and solved for C_R_^*^, general relationships of

$$C_{\text{R}}{}^{*}=C_{R}\cdot 7.692\cdot {\text{e}}^{-2.028\cdot \theta \text{rel}}$$

and

$$C_{\text{R}}{}^{*}=C_{R}\cdot 4.107\cdot {\text{e}}^{-1.392\cdot \theta \text{rel}}$$

are obtained for maize and soybean, respectively. These empirical equations were used to calculate C_R_^*^ for each C_*R*_ and associated θ_rel_ value detected in the field trials in order to monitor temporal changes in plant root activity under variable soil moisture conditions.

**Table 2:** Effects of relative water saturation of soil around the root system (θ_rel_), plant age (days) and species identity on the relative root electrical capacitance (C_rel_) of maize and soybean in a pot experiment based on linear mixed effects models.

**Variables and effects**	**d.f**.	***F***	**p**
***C**_*rel*_**For The Two Species In The Pot Experiment***			
Intercept	1,266	3921.14	<**0.001**
θ_rel_	1,266	3139.57	<**0.001**
Plant age	1,26	0.12	0.736
Species	1,26	121.42	<**0.001**
θ_rel_ × Plant age	1,266	0.16	0.691
θ_rel_ × Species	1,266	107.45	<**0.001**
Plant age × Species	1,26	3.67	0.067
θ_rel_ × Plant age × Species	1,266	1.12	0.290
***C**_*rel*_**for maize in the pot experiment***			
Intercept	1,133	2116.89	<**0.001**
θ_rel_	1,133	1665.62	<**0.001**
Plant age	1,13	2.31	0.153
θ_rel_ × Plant age	1,133	0.57	0.451
***C**_*rel*_**for soybean in the pot experiment***			
Intercept	1,133	1912.17	<**0.001**
θ_rel_	1,133	1466.93	<**0.001**
Plant age	1,13	1.36	0.264
θ_rel_ × Plant age	1,133	0.54	0.464

*Significant differences (p < 0.05) are shown in bold*.

### Field trial A–maize

Measurements carried out in the maize field demonstrated a high temporal variation in θ_rel_ with means ranging between 0.214 on DAS 106 and 0.547 on DAS 123 (Table 3), and relatively high spatial heterogeneity within measurement days with coefficients of variation (CV) ranging from 8.2 to 12.5%. Due to the spatially variable SWC and differences in root system size between sample plants, the value of C_*R*_ recorded at each measurement time also varied over a wide range (CV 8.1–26.1%). ANOVA revealed no significant differences in θ_rel_ and C_*R*_ between the three replicate row-segments at any measurement time, so the data were pooled (*n* = 48) for further analysis. Regression analysis revealed a significant exponential relationship between θ_rel_ and C_*R*_ (*n* = 50; *R*^2^ = 0.496–0.663; *p* < 0.01) for the data of each measurement event.

**Table 3:** Relative water saturation of soil around the root system (θ_rel_), measured root electrical capacitance (C_*R*_ in nanofarads, nF) and phenological stage of maize on the BBCH-scale (Meier, 2001) at different measurement times (DAS: days after sowing).

**DAS**	**θ_rel_**	**C_*R*_ (nF)**	**Phenological stage [*BBCH code*]**
	**mean ± SD**	**mean ± SD**	
36	0.516 ± 0.054	4.71 ± 0.86	4 leaves unfolded [*14*]
50	0.488 ± 0.045	15.09 ± 2.78	7-8 leaves unfolded [*17*]
64	0.236 ± 0.023	13.74 ± 2.60	9-10 leaves unfolded [*19*]
78	0.537 ± 0.067	34.46 ± 3.84	Beginning of pollen shedding, stigmata visible [*63*]
92	0.338 ± 0.033	25.02 ± 3.63	Flowering completed, stigmata drying [*67*]
106	0.214 ± 0.018	16.24 ± 1.73	Early milk [*73*]
123	0.547 ± 0.052	19.28 ± 2.76	Early dough [*83*]
134	0.237 ± 0.023	7.06 ± 0.78	Physiological maturity [*87*]
147	0.266 ± 0.023	5.24 ± 1.37	Fully ripe: kernels hard and shiny [*89*]
161	0.521 ± 0.041	2.35 ± 0.41	Plant dead and collapsing [*97*]

**Figure 4:**
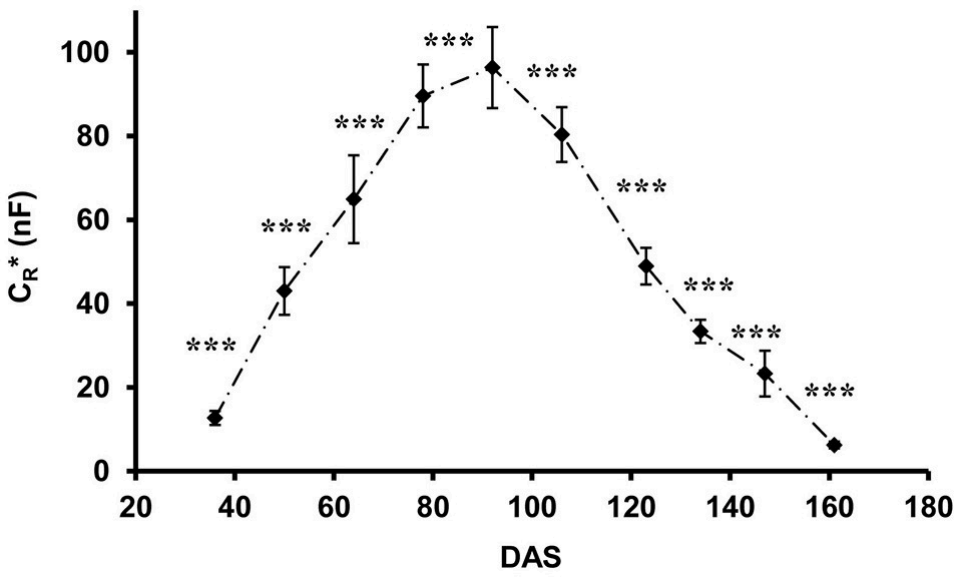
Apparent root electrical capacitance (C_R_^*^ in nanofarads, nF) related to time (DAS: days after sowing) in maize plants. Vertical bars show standard deviations (*n* = 48). Asterisks indicate the statistical differences between the results of the two consecutive measurements (***p < 0.001).

C_R_^*^, calculated from field data using Equation 3, showed characteristic temporal changes during plant ontogeny (Figure 4), with values increasing continuously from 12.7 ± 1.7 nF (mean ± SD; *n* = 48) at the 4-leaf stage (DAS 36) to 96.4 ± 9.7 nF when flowering was completed (DAS 92), thereafter decreasing to 6.3 ± 0.8 nF at the end of the experiment (DAS 161; plant dead and collapsing). Statistical analysis (*t*-test) showed highly significant (*p* < 0.001) changes in C_R_^*^ between any two consecutive measurement times.

### Field trial B–soybean

In the soybean field, the mean θ_rel_ fluctuated over time between 0.242 on DAS 54 and 0.746 on DAS 162, a day after heavy rain (Table 4), with lower spatial heterogeneity (CV of 3.9–10.7%) than was observed in the maize field. ANOVA showed no significant differences in θ_rel_ and C_*R*_ between the three replicate plots of either the *CON* or *INO* treatments for any measurement time, so the data were subsequently pooled (*n* = 48). Unpaired *t*-tests revealed no significant differences between the pooled data groups of the *CON* and *INO* treatments for θ_rel_ throughout the experiment. The more homogeneous distribution of SWC resulted in less variable C_*R*_ (CV 6.2–12.3%) compared to that of maize plants. For both treatments, significant exponential relationships were found between C_*R*_ and θ_rel_ (*n* = 48; *R*^2^ = 0.383–0.710; *p* < 0.01) for the data recorded at each measurement time (results not shown).

**Table 4:** Relative water saturation of soil around the root system (θ_rel_), measured root electrical capacitance (C_*R*_ in nanofarads, nF) and phenological stage of control (*CON*) and AMF-inoculated (*INO*) soybean plants on the BBCH-scale (Meier, 2001) at different measurement times (DAS: days after sowing).

**DAS**	**CON**		**INO**		**Phenological stage [*BBCH code*]**
	**θ_rel_**	**C_*R*_ (nF)**	**θ_rel_**	**C_*R*_ (nF)**	
	**mean ± SD**	**mean ± SD**	**mean ± SD**	**mean ± SD**	
39	0.413 ± 0.033	2.00 ± 0.12	0.415 ± 0.031	2.01 ± 0.13	Leaves on the 2nd node unfolded [*12*]
54	0.245 ± 0.018	2.47 ± 0.26	0.242 ± 0.019	2.58 ± 0.27	Leaves on the 4th node unfolded [*14*]
69	0.401 ± 0.036	5.21 ± 0.46	0.400 ± 0.032	5.35 ± 0.42	First flowers opened [*60*]
83	0.354 ± 0.038	8.77 ± 0.69	0.356 ± 0.033	8.98 ± 0.70	Main period of flowering [*65*]
97	0.319 ± 0.025	8.25 ± 0.64	0.325 ± 0.022	8.41 ± 0.63	Beginning of pod filling [*73*]
113	0.632 ± 0.037	12.36 ± 1.05	0.641 ± 0.034	12.70 ± 1.28	Continuation of pod filling [*75*]
125	0.278 ± 0.021	6.93 ± 0.53	0.276 ± 0.024	6.99 ± 0.55	Advanced pod filling [*77*]
139	0.326 ± 0.028	4.81 ± 0.58	0.330 ± 0.024	4.86 ± 0.56	Beginning of pod and seed ripening [*81*]
150	0.655 ± 0.038	3.55 ± 0.30	0.663 ± 0.031	3.61 ± 0.29	Main period of pod and seed ripening [*85*]
162	0.743 ± 0.031	2.14 ± 0.26	0.746 ± 0.029	2.19 ± 0.27	Pods are ripe, beans dry and hard [*89*]

**Figure 5:**
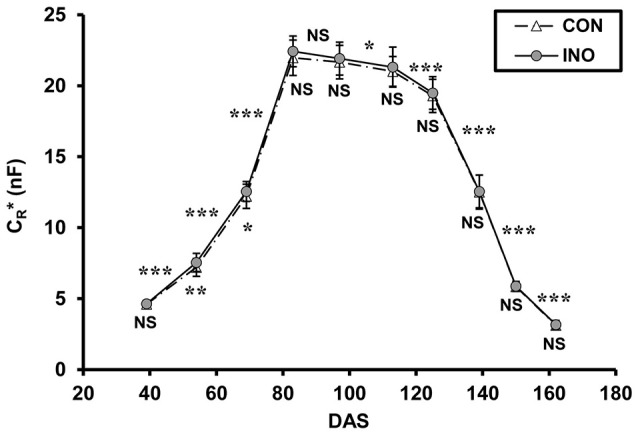
Apparent root electrical capacitance (C_R_^*^ in nanofarads, nF) related to time (DAS: days after sowing) in control (*CON*) and AMF-inoculated (*INO*) soybean plants. Vertical bars show standard deviations (*n* = 48). Asterisks above the curves indicate statistical differences between the results of two consecutive measurements (^***^, ^**^, ^*^, NS: *p* < 0.001, 0.01, 0.05, non-significant, respectively). Asterisks below the data markers refer to differences between *CON* and *INO* groups.

Mean C_R_^*^, calculated using Equation 4, showed similar phenological changes for the two treatments, but the pattern was somewhat different from that of maize (Figure 5). After the first measurement (DAS 39; 2-node stage), when C_R_^*^ values of 4.61 ± 0.20 nF (mean ± SD; *n* = 48) and 4.63 ± 0.22 nF were recorded for *CON* and *INO* plants, respectively, this indicator of root activity increased sharply during the vegetative phase, reaching a peak at the main period of flowering (DAS 83; *CON*: 21.97 ± 1.25 nF; *INO*: 22.42 ± 1.08 nF). Thereafter, it steadily but slowly decreased during pod filling till DAS 113 (with no significant change between DAS 83 and 97), then sharply decreased throughout the seed-ripening period to 3.12 ± 0.28 nF (*CON*) and 3.17 ± 0.29 nF (*INO*) at the end of the experiment (DAS 162). The effect of AMF treatment was visible in the root capacitance response (Figure 5): according to *t*-tests, the mean C_R_^*^ of AMF-inoculated plants was significantly higher than that of the control at the 4-node stage (DAS 54; *CON*: 7.20 ± 0.63 nF; *INO*: 7.55 ± 0.64 nF; *p* < 0.01) and at the beginning of flowering (DAS 69; *CON*: 12.21 ± 0.85 nF; *INO*: 12.55 ± 0.70 nF; *p* < 0.05), while borderline significance was observed at the main flowering period (DAS 83; *p* = 0.063).

**Figure 6:**
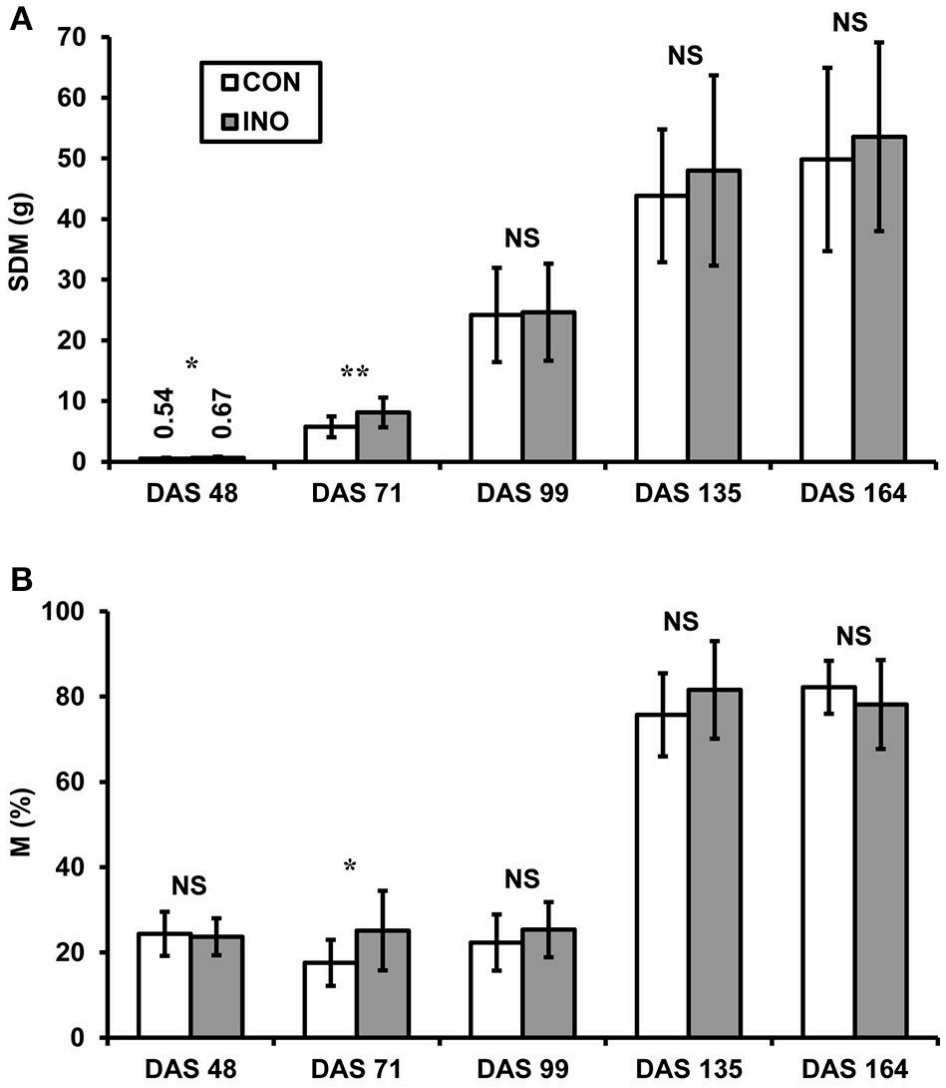
**(A)** Shoot dry mass (SDM) and **(B)** intensity of AMF colonization (M%) of control (*CON*) and AMF-inoculated (*INO*) soybean plants at different sampling times (DAS: days after sowing). Vertical bars show standard deviations. Asterisks indicate statistical differences between *CON* and *INO* groups (^**^, ^*^, NS: *p* < 0.01, 0.05, non-significant, respectively).

AMF-inoculated plants exhibited greater SDM than the control ones at each harvest time (Figure 6A), but the difference was only significant for the data from the first two sampling events in the vegetative and early flowering period (DAS 48 and 71). At the final harvest, SDM was 49.8 ± 15.1 g (*n* = 30) for *CON* and 53.6 ± 15.6 g for *INO* plants, including 19.5 ± 6.4 g and 19.2 ± 6.2 g dry grain, respectively (not significant). Microscopic observation of the roots showed a relatively low intensity of AMF colonization (M_*mean*_ = 18.1–25.4%) till DAS 99, with significantly higher mean value for inoculated plants than for the controls only at the second harvest (Figure 6B). The percent AMF colonization increased considerably during pod filling up to 82.2 ± 6.2% and 78.2 ± 10.4% for *CON* and *INO* plants, respectively, with no significant differences between the treatments.

## Discussion

The pot experiment demonstrated a nearly linear increase in C_*S*_ with SWC. The reason is that ε_*r*_ of water is much higher (~80 at 1 kHz) than that of the other main soil constituents, including air (ε_*r*_ ~1) and mineral solids (ε_*r*_ < 5), so rising SWC leads to an increase in the ε_*r*_ of the moistened soil and accordingly in its capacitance (Hilhorst, 1998). In each case, C_*S*_ proved to be substantially higher than the corresponding C_*R*_, meeting the basic criterion of the two-dielectric capacitor model (Rajkai et al., 2005; Dietrich et al., 2013) and confirming the dominance of the root tissue in determining C_*R*_. Moreover, the results imply that the increase in C_*R*_ in relation to θ_rel_ is not attributable to rising C_*S*_; consequently, C_*S*_ data are not really informative for C_*R*_ determination. These findings on the exponential relationship of C_*R*_ to θ_rel_ are consistent with those of Dalton (1995), who reported similar results for a single tomato plant grown in a progressively drying sandy soil. Dalton suggested that the reduction in SWC reduced the root surface area contacted with the soil solution, resulting in a proportional decline in C_*R*_. In contrast, the decrease in C_*R*_ with SWC has also been attributed to the increased electrical resistance of the ground electrode–soil interface (Ellis et al., 2013b) or, as stated by Dietrich et al. (2012), to “less effective contact between the plant and the root–soil solution menisci distributed along the root surface.”

The parameters of C_rel_-θ_rel_ functions proved to be independent of plant age. This greatly simplifies the application of the method, as a general species-specific function can be used irrespective of plant phenological stage and RSS. The strong species dependence of the relationship between C_rel_ and θ_rel_ is in accordance with the species- and substrate-specific nature of the C_*R*_–RSS relationships reported in previous papers (Chloupek et al., 2010) and also found in the present study (Figure 1). The positive intercept derives from electrode polarization and the capacitance of the plant stem base (Cseresnyés et al., 2016b), and it is thought to be a function of SWC (Ozier-Lafontaine and Bajazet, 2005; McBride et al., 2008). The changes in C_rel_ in relation to θ_rel_ for a given species are also likely to be influenced by the soil type, but further investigations are required to assess this effect.

Field trials clearly demonstrated characteristic phenological changes in C_R_^*^, as an indicator of root activity. The maize root system was observed to attain maximum activity in the flowering period, after which a sharp decrease was observable. This is in agreement with previous findings on plant morphological and physiological changes, studied with a variety of methods. Due to its determinate growth habit, the maize growth rate approaches zero at the beginning of flowering (Zegada-Lizarazu et al., 2012). A phenological study showed a decrease in whole-plant transpiration and water and ion uptake from the main flowering stage to grain-filling and plant maturity (Novák and Vidovič, 2003). Consequently, maize root length density and leaf area were observed to show similar temporal patterns during crop development, with sigmoid growth until pollen shedding followed by a decrease (Liedgens and Richner, 2001). The reduction in leaf area is the consequence of foliar senescence, which accelerates as the plant approaches physiological maturity, leading to a decrease in canopy transpiration and root water uptake (Tsimba et al., 2013). Moreover, the transpiration rate per unit surface area of green leaves was also observed to decrease during the late generative phases (Medrano et al., 2005). By focusing on root development, Gao et al. (1998) reported that both the relative proportion and the uptake activity of absorptive, apical young root segments decreased during plant maturity with a concurrently increasing ratio of older roots (primarily responsible for transport), resulting in the declining water uptake of the root system.

A soybean study demonstrated that plant leaf area and daily transpiration increased until the flowering period and then declined during the maturity stages (Setiyono et al., 2008). Both growth chamber and field data showed a trend of decreasing hydraulic conductance and photosynthetic activity in aging soybean leaves (Locke and Ort, 2014), which was obviously associated with a reduced root water uptake rate.

The root activity in both crops was found to be the highest during the main flowering period but, unlike that of maize, the C_R_^*^ calculated for soybean remained almost unchanged and then decreased only slightly over the one-month pod-filling period. This is probably attributable to the indeterminate growth habit of the soybean cultivar used in the present study. In indeterminate varieties, the elongation of the main stem and the development of side shoots continue parallel to the formation of inflorescences and photosynthetically active leaves. This compensates for leaf senescence for a while, maintaining a high level of root system activity throughout the pod-filling stages. This is consistent with previous studies indicating that the green leaf area of indeterminate soybean cultivars was constantly high during the weeks of flower formation, followed by a continuous decline due to increasing leaf senescence (Hida et al., 1995). Other authors found that the growth of soybean roots continued during pod-filling and seed ripening (Torrion et al., 2012). Nevertheless, suberin deposition, root senescence and declined activity caused a reduction in C_*R*_ in aging root populations (Dalton, 1995; Ellis et al., 2013a; Cseresnyés et al., 2016a).

Capacitance measurements carried out at the late vegetative and early flowering periods of soybean ontogeny (DAS 54 and 69) showed significantly higher root activity for AMF-inoculated plants than for the controls. This was confirmed by the plant harvest and the microscopic investigation of isolated roots, indicating significantly higher SDM (on DAS 48 and 71) and intensity of AMF colonization (on DAS 71) in inoculated plants. Irrespective of the AMF treatment, root colonization proved to be moderate (18–25%) until pod filling, and then rapidly increased up to seed ripening. A previous work by Liang et al. (2015) revealed similar intraradical development of mycorrhizae in field-grown soybean varieties, with the highest colonization rate (55–65%) in the maturity stages. Several studies reported altered root architecture, increases in photosynthetic rate, transpiration rate, stomatal and root conductance, as well as enhanced plant growth and water and nutrient uptake in response to AMF symbiosis (Augé, 2001). Nevertheless, no convincing evidence was found to suggest that AMF inoculation improved host plant biomass. Therefore, the enhanced plant biomass was presumably due to the nutrient and additive content of the commercial product used for inoculation rather than to the symbiotic relationship.

The results of this study suggest that the calculation of apparent root capacitance (C_R_^*^) using empirically established C_rel_-θ_rel_ functions is a suitable approach for the *in situ* monitoring of the seasonal pattern of root system activity under real field conditions as a function of environmental factors affecting plant growth (e.g., AMF inoculation). Importantly, capacitance data are of a relative nature, from which it is difficult to calculate the absorbing RSA or the actual root water uptake rate (as a volume per time unit) directly. Moreover, one drawback of the capacitance method over conventional procedures i.e., rhizotrons or core sampling, is that it cannot visualize root architecture, penetration depth or distribution pattern (Cseresnyés et al., 2016a). In the case of deep-rooting dicot plants, including soybean, the signal loss of the capacitance response is assumed to be higher (Ellis et al., 2013a). This may change the relationship between C_*R*_ and RSS, affecting the accuracy of root size evaluation. In field studies carried out on relatively large areas, the spatial heterogeneity of soil properties (e.g., texture or organic matter content) may also cause a bias in the C_*R*_ detected.

Nevertheless, the capacitance method offers several practical advantages over other techniques. The measurements are simple, inexpensive, time-saving and, most importantly, do not necessitate destructive sampling, plant disposal or area disturbance; in addition, they are not confounded by roots of neighboring plants. Furthermore, this *in situ* technique allows changes in the root development of the same plants to be followed on a relatively fine time-scale. This rapid process enables a large number of plants to be quickly measured under field conditions. Finally, and perhaps most importantly, the method presented here provides useful insights into the functional aspect of root development, as the magnitude of C_*R*_ incorporates the extension and actual activity of the plant root system (Dalton, 1995; Cseresnyés et al., 2016a).

It was concluded that the strong effect of SWC on C_*R*_ can be considered the most influential constraint on the applicability of the capacitance method, particularly under field conditions. However, the present work suggests that the root capacitance method could be usefully adapted for time course studies on root activity in the field, and for comparing single-time capacitance data collected in areas with spatially heterogeneous soil water status.

The measurement of root electrical capacitance in field-grown crops is potentially of benefit for a diverse range of basic and applied research in agriculture. The method could facilitate the selection of genotypes with greater RSS and could thus lead to the successful production of cultivars with increased grain yield. The knowledge of root activity over time may serve to reveal differences in plant responses to stressful weather and soil conditions (e.g., drought, temperature anomalies, nutrient deficiencies), biotic stresses (e.g., pathogenic infections, weed competition) or agricultural practices (e.g., soil tillage, plant nutrition, herbicide application). Due to its versatile adaptability, capacitance measurement could partially substitute for or be integrated with the widely used intrusive techniques. Therefore, notwithstanding the drawbacks and limitations that should be taken into account when using this method, it will be beneficial in future field studies.

## Author contributions

IC designed and conducted pot experiment and capacitance measurements, discussed the results and wrote the paper. KS analyzed the data. KR supervised the project and helped in data interpretation. AF, PM, and RK designed and carried out the field trials and plant investigations. TT designed and supervised soybean experiment and helped in writing paper. All authors read the manuscript and approved the submission.

### Conflict of interest statement

The authors declare that the research was conducted in the absence of any commercial or financial relationships that could be construed as a potential conflict of interest.

## Abbreviations

AC: Alternating current
AMF: Arbuscular mycorrhizal fungi
C_*R*_: Root electrical capacitance
C_*R*_^*^: Apparent root electrical capacitance
C_rel_: Relative root electrical capacitance
C_*S*_: Soil electrical capacitance
LME: Linear mixed effect models
RDM: Root dry mass
RL: Root length
RSA: Root surface area
RSS: Root system size
SDM: Shoot dry mass
SWC: Soil water content.
